# Deep learning-enhanced detection of dental restorations and orthodontic appliances in panoramic radiographs using a clinically annotated dataset

**DOI:** 10.2340/aos.v85.46619

**Published:** 2026-08-19

**Authors:** Le-Cun Xiao, Hao-Ran Zhao, Ning Zhao, Yao-Xiang Xu, Wen-Lin Xiao

**Affiliations:** a School of Computer Science and Engineering, The University of New South Wales, Sydney, Australia; b Department of Oral and Maxillofacial Surgery, The Affiliated Hospital of Qingdao University, Qingdao, China; c School of Stomatology, Qingdao University, Qingdao, China

**Keywords:** Deep learning, panoramic radiography, object detection, faster R-CNN, dental restorations, medical imaging

## Abstract

**Objective:**

Manual interpretation of dental panoramic radiographs is labor intensive and prone to diagnostic fatigue, particularly in high-volume settings. While artificial intelligence offers potential solutions, existing automated detection models often suffer from limited generalization due to small-scale, inconsistent datasets and scale mismatches between generic algorithms and dental structures. This study aimed to develop a deep learning framework trained on a clinically annotated retrospective dataset to improve the multiclass detection accuracy of dental restorations and appliances.

**Material and method:**

A retrospective dataset comprising 2,434 anonymized panoramic X-ray images was curated and annotated by trained dental professionals for six categories: crowns, root canal filling, fillings, bridges, implants, and braces. The baseline dataset used for contextual comparison had a different annotation scope and class composition from the enhanced dataset; therefore, it was used only as a descriptive reference rather than as a directly matched comparator. A faster Region based Convolutional Neural Network (R-CNN) model with a ResNet-50 Feature Pyramid Network backbone was trained using domain-specific optimizations, including customized anchor resizing (16–256 pixels) and adaptive data augmentation.

**Findings:**

The enhanced dataset setting achieved an mAP@0.5 of 0.75, which was numerically higher than the baseline setting but should not be interpreted as a directly controlled improvement because the datasets differed in source and annotation scope. The system demonstrated significant gains in detecting small targets, with average recall increasing from 0.59 to 0.81. Performance varied by category; implants achieved the highest precision at 0.93, while braces exhibited lower precision at 0.32 due to overlapping artifacts. The average processing time was under 1.2 s per image on the tested laptop workstation.

**Conclusion:**

These findings suggest that clinically curated data and domain-adaptive design may improve detection performance in panoramic radiography. The framework may have potential as a decision-support tool, but further external and multicenter validation is needed before clinical deployment.

## Introduction

Dental X-ray imaging has long served as a cornerstone of clinical dental practice, offering irreplaceable diagnostic value, cost-effectiveness, and accessibility in detecting structural restorations and appliances such as dental caries, root canal filling treatments, and implants [1]. However, manual interpretation of these images remains a labor-intensive and error-prone task, particularly in high-volume clinical settings. Prolonged exposure to repetitive visual patterns may lead to diagnostic fatigue, elevating the risk of missed or misdiagnosed cases – a phenomenon corroborated by the practical experiences of dental practitioners [2]. These limitations underscore the urgent need for automated solutions to augment human expertise and enhance diagnostic reliability.

Recent advancements in artificial intelligence (AI), particularly breakthroughs in deep learning, have revolutionized medical image analysis. Convolutional neural networks (CNNs) and object detection frameworks such as faster R-CNN, YOLO, and RetinaNet have demonstrated remarkable efficacy in diverse tasks, ranging from detecting pulmonary nodules and quantifying blood cells in microscopic slides to identifying tuberculosis progression in lung imaging [3–5]. In the dental domain, AI models have been applied to tooth segmentation, caries detection, and periodontal condition assessment, achieving performance levels comparable to those of experienced clinicians [6, 7]. Notably, two-stage detectors (e.g. faster R-CNN) excel in scenarios requiring high precision, such as detecting subtle or overlapping anatomical structures, rendering them particularly suitable for dental X-ray analysis [8].

Leveraging its region proposal network (RPN) and flexible backbone architecture, Faster R-CNN has gained increasing traction in dental imaging applications. Previous studies have employed this framework to detect restorations, root canal filling, and implants in panoramic radiographs, reporting promising outcomes [9, 10]. For instance, Lee et al. achieved a mean average precision (mAP) of 85% in dental restoration detection by integrating faster R-CNN with image enhancement techniques [11]. Despite these advancements, existing models often suffer from limited generalization and accuracy due to insufficient or suboptimal training data. Many studies rely on small-scale, inconsistently annotated datasets, resulting in suboptimal performance (e.g. mAP@0.5 ≈ 0.50) and restricted clinical applicability [12].

Despite preliminary advancements in AI for dental image analysis, existing research continues to face critical challenges: limited model generalization due to small-scale datasets and annotation inaccuracies, compounded by the technical complexity of multiclass restorations and appliances detection in dental radiographs, such as small-scale targets and background interference. To address these challenges, this study developed an automated detection system through the construction of a clinically annotated panoramic X-ray dataset and optimization of a domain-adaptive detection framework. By using standardized annotation and multiscale feature extraction, the system was developed to improve detection performance in complex scenarios. Its potential clinical utility warrants further evaluation in external datasets and real-world workflows.

Although several previous studies have reported higher performance metrics in dental image analysis, direct comparison with the present study should be made cautiously because of differences in imaging modality, target definition, category granularity, dataset composition, and evaluation metrics. Some prior studies focused on single or fewer categories, broader grouped labels, tooth numbering, classification, segmentation, or nonpanoramic images, whereas the present study evaluated six categories of dental restorations and orthodontic appliances on panoramic radiographs, including challenging classes such as fillings and braces. Therefore, this study is positioned not as a direct performance benchmark against prior models but as a clinically annotated multiclass detection study that reports class-wise heterogeneity and limitations in a real-world panoramic radiography setting.

## Methods

### Dataset collection and annotation

The study utilized a dataset of 2,434 panoramic dental X-ray images retrospectively collected from routine clinical examinations conducted at the Radiology Department of the Affiliated Hospital of Qingdao University. The radiographs were acquired during routine clinical care between January 2020 and August 2024. Image collection was completed in August 2024, and dataset annotation was initiated concurrently and finalized in April 2025. All images were fully anonymized prior to annotation and subsequent analysis. Formal ethical approval for the use of these retrospective, de-identified data in research was obtained prior to model training and performance evaluation.

All images were manually annotated by trained dental professionals using Labelme, an open-source annotation tool, to delineate six categories of dental restorations and orthodontic appliances: crowns, root canal filling, fillings, bridges, implants, and braces. Initial bounding-box annotations were performed by the author responsible for data curation and were subsequently reviewed by the author responsible for validation. Both annotators had dental training and were familiar with the radiographic appearance of dental restorations and orthodontic appliances on panoramic radiographs. Disagreements or uncertain annotations were resolved through consensus discussion with a senior supervising dental investigator. To assess annotation consistency, 10% of the original unlabelled radiographs (*n* = 244) were randomly selected as a quality-control subset and independently annotated by a second trained dental professional. The independently generated bounding-box annotations were compared with the initial annotations at the object level, and mean inter-annotator Intersection over Union (IoU) was calculated across matched annotations. The mean inter-annotator IoU was 0.92, indicating high spatial consistency between annotators. Cases with low overlap or category disagreement were re-examined and resolved through consensus discussion. The dataset exhibited moderate class imbalance, with fillings and crowns being more prevalent than braces or implants. The dataset was divided into training, validation, and test sets containing 1,944, 246, and 244 radiographs, respectively, while maintaining balanced class distributions across subsets.

The distribution of object-level annotations in the enhanced dataset across the training, validation, and test sets is summarized in Table 1. Annotation counts refer to bounding-box annotations rather than unique radiographs, because each panoramic radiograph could contain multiple restorations or appliances. The baseline dataset was derived from the public dental-X-ray-analysis project, and its composition is summarized in Table 2. It included 1,269 labeled dental radiographs, with 1,075, 121, and 73 images in the training, validation, and test sets, respectively. The baseline dataset contained 9,283 object-level bounding-box annotations across four categories: cavities, fillings, impacted teeth, and implants. The enhanced dataset used in the present study differed from the baseline dataset in source, annotation scope, category definitions, and class composition. Therefore, the baseline dataset was used only as a contextual descriptive reference rather than as a directly matched control, and no formal inferential comparison was performed between the two datasets.

**Table 1:** Distribution of object-level annotations across the training, validation, and test sets.

Class	Training	Validation	Test	Total, *n* (%)
Crown	2,507	278	325	3,110 (29.1%)
Root canal filling	1,726	185	204	2,115 (19.8%)
Fillings	3,921	489	476	4,886 (45.7%)
Bridge	216	32	39	287 (2.7%)
Implant	128	15	42	185 (1.7%)
Braces	73	18	27	118 (1.1%)
Total	8,571	1,017	1,113	10,701 (100%)

**Table 2:** Distribution of object-level bounding-box annotations in the baseline dataset.

Class	Training	Validation	Test	Total, *n* (%)
Cavity	576	43	22	641 (6.9%)
Fillings	5,242	540	315	6,097 (65.7%)
Impacted tooth	428	38	32	498 (5.4%)
Implant	1,784	159	104	2,047 (22.0%)
Total	8,030	780	473	9,283 (100%)

### Ethics approval and consent to participate

This study was approved by the Institutional Review Board of Qingdao University (approval No. QYFY WZLL 30194). The study involved retrospective analysis of anonymized panoramic radiographs that had been acquired during routine clinical care prior to study initiation. Ethical approval was obtained before any research-related data analysis was conducted. Written informed consent was obtained from all participants (or from a parent/legal guardian for minors). All panoramic radiographs were anonymized before analysis. All methods were performed in accordance with the Declaration of Helsinki and relevant guidelines and regulations. Written informed consent to publish clinical details and images was obtained from all participants or their legal guardians.

### Model architecture

The detection framework was based on faster R-CNN with a ResNet-50 backbone integrated with a Feature Pyramid Network (FPN) to enhance multiscale feature extraction. Faster R-CNN was selected as an established two-stage object-detection framework because its region-proposal mechanism and FPN-based multiscale feature representation are well suited for detecting small, overlapping, and anatomically complex targets in panoramic radiographs. Although newer one-stage or transformer-based detectors may offer advantages in speed or general-purpose object detection, their superiority is not guaranteed for small-object detection in dental panoramic images without task-specific benchmarking. Therefore, this study used faster R-CNN as a robust and widely validated framework that allowed anchor customization and comparison with previous dental panoramic radiography studies [8, 13–15]. To adapt the model to dental imaging characteristics, anchor sizes were customized to {16, 32, 64, 128, 256} pixels, and aspect ratios were set to {0.5, 1.0, 2.0}. Training employed the AdamW optimizer with a weight decay of 1×10^-4^ and an initial learning rate of 1×10^-4^, which was reduced by 50% upon validation mAP plateau. Due to GPU memory constraints, gradient accumulation over four iterations simulated an effective batch size of 8. Data augmentation strategies included random rotation (±10°), horizontal flipping (50% probability), brightness adjustment (±20%), CutMix, and MixUp to improve generalization.

### Evaluation metrics

Model performance was evaluated using mAP at an IoU threshold of 0.5 (mAP@0.5) as the primary metric, complemented by precision, recall, F1-score, and average recall at 100 proposals (AR@100). Implementation leveraged PyTorch and Torchvision libraries, with reproducibility ensured through version-controlled code and fixed random seeds. Inference time was measured with a batch size of 1 using a laptop workstation equipped with an Intel Core i9-14900HX CPU, 16 GB RAM, and an NVIDIA GeForce RTX 4060 Laptop GPU with 8 GB VRAM. The reported inference time represents the average per-image processing time during model evaluation.

### Statistical analysis

Analyses were performed per image with patient-wise train/validation/test splits to reduce data leakage. Model performance was evaluated descriptively using mAP@0.5, AR@100, precision, recall, and F1-score, reported overall and per class where applicable. Per-class TP, FP, and FN counts were reported to support interpretation of class-wise performance and class imbalance. Because the baseline and enhanced datasets differed in annotation scope, target categories, and class composition, formal inferential comparisons between datasets were not performed; results were interpreted descriptively. Statistics and plotting were performed using Python (NumPy, SciPy, and scikit-learn), and deep learning was implemented in PyTorch.

## Findings

### Descriptive dataset-level performance

The performance of the proposed model was evaluated across two datasets: a baseline dataset with limited annotations and an enhanced dataset comprising 2,434 clinically annotated panoramic X-ray images. As shown in Table 3, the enhanced dataset was associated with higher dataset-level detection metrics, achieving an mAP@0.5 of 0.75 compared to the baseline dataset (mAP@0.5 = 0.50). Average recall (AR@100) increased from 0.59 to 0.81, suggesting improved sensitivity to rare and small-scale restorations and appliances such as root canal filling and fillings. Due to the difference in category composition between the baseline and enhanced datasets, direct class-wise percentage comparison is not applicable. Therefore, performance is reported separately for each dataset using per-class Precision, Recall, and F1-score (Table 4 for the baseline dataset and Table 5 for the enhanced dataset), together with the macro-averaged results. As shown in Table 3 and Table 6, the enhanced dataset achieves higher macro-average recall and F1-score, suggesting better overall performance within this experimental setting. In addition, dataset-level metrics such as mAP@0.5 and AR@100 are reported to provide a descriptive summary of performance across different dataset configurations.

**Table 3:** Descriptive dataset-level performance of the baseline and enhanced datasets.

Metric	Baseline dataset	Enhanced dataset
mAP@0.5	0.50	0.75
AR@100	0.59	0.81

**Table 4:** Per-class detection performance on the baseline dataset.

Class	Precision	Recall	F1	TP	FP	FN
Implants	0.60	0.55	0.57	57	38	47
Fillings	0.61	0.79	0.69	252	168	63
Impacted teeth	0.66	0.69	0.67	22	12	10
Cavity	0.42	0.56	0.49	12	15	10
Macro average	0.57	0.65	0.6			

**Table 5:** Per-class detection performance on the enhanced dataset.

Class	Precision	Recall	F1	TP	FP	FN
Crowns	0.82	0.86	0.84	280	62	45
Fillings	0.74	0.56	0.64	114	40	90
Root canal filling	0.77	0.80	0.78	381	114	95
Bridges	0.70	0.68	0.69	27	12	12
Implants	0.93	0.92	0.92	39	3	3
Braces	0.32	0.89	0.45	21	45	3
Macro average	0.71	0.76	0.72			

**Table 6:** Performance comparison before and after anchor tuning.

Class	Precision (default / custom)	Recall (default / custom)	F1-score (default / custom)
Crowns	0.81/0.82	0.84/0.86	0.83/0.84
Fillings	0.73/0.74	0.53/0.56	0.62/0.64
Root canal filling	0.75/0.77	0.78/0.80	0.76/0.78
Bridges	0.67/0.70	0.62/0.68	0.64/0.69
Implants	0.93/0.93	0.88/0.89	0.90/0.92
Braces	0.29/0.32	0.79/0.89	0.42/0.45
Macro average	0.70/0.71	0.74/0.76	0.70/0.72

### Impact of anchor optimization

**Figure 1:**
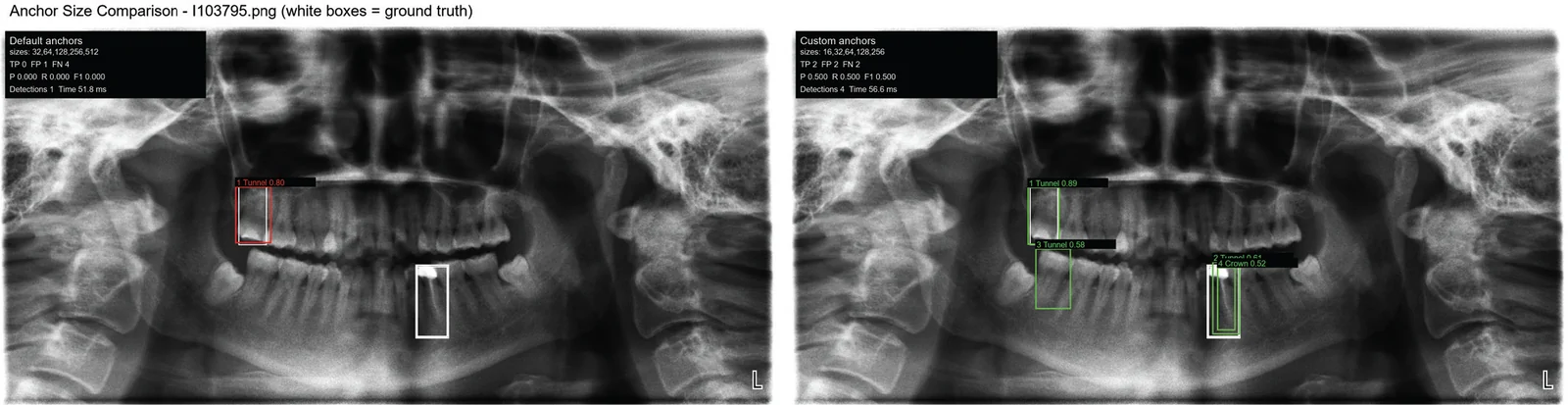
Improved small-target detection with custom anchor sizes.

Custom anchor sizes (16–256 pixels) were introduced to address the scale mismatch between natural image defaults (32–512 pixels) and dental restorations and appliances. Anchor tuning yielded modest improvements in overall performance. Specifically, the mAP@0.5 increased from 0.70 to 0.71 based on the training evaluation outputs, while the macro-average recall increased from 0.74 to 0.76, as summarized in Table 6. Small gains were observed for fillings and root canal filling following this adjustment, with fillings’ recall improving by 5% and root canal filling’ precision by 2.7%, respectively. These results are illustrated in Figure 1, where white boxes indicate ground-truth annotations, red boxes indicate predictions generated using the default anchor configuration, and green boxes indicate predictions generated using the custom anchor configuration.

### Target size distribution and anchor configuration

To quantitatively justify the anchor size configuration, we analyzed the distribution of ground-truth bounding box sizes derived from the LabelMe annotations. As summarized in Table 7, the localized restoration and implant categories, including crowns, fillings, root canal filling, bridges, and implants, showed median long-edge sizes of approximately 165–221 pixels, with most instances falling within or near the custom anchor range. In contrast, braces showed substantially larger long-edge sizes because they often extended across multiple teeth or dental arches. Therefore, the custom anchor configuration was primarily designed to improve proposal generation for smaller localized targets, while braces represented a distinct elongated target category requiring separate interpretation.

**Table 7:** Per-class bounding box size statistics in the training set.

Class	P10	P25	P50	P75	P90	P95
Crowns	143.6	157.7	173.1	188.5	203.8	212.6
Fillings	142.3	157.3	170.9	185.4	200.0	210.0
Root canal filling	136.9	151.5	165.4	179.6	193.8	202.9
Bridges	182.1	200.0	221.2	248.6	286.8	305.8
Implants	111.1	132.0	175.0	200.3	227.0	243.4
Braces	522.4	667.6	742.3	832.1	914.9	965.9

### Class-wise detection performance

**Figure 2:**
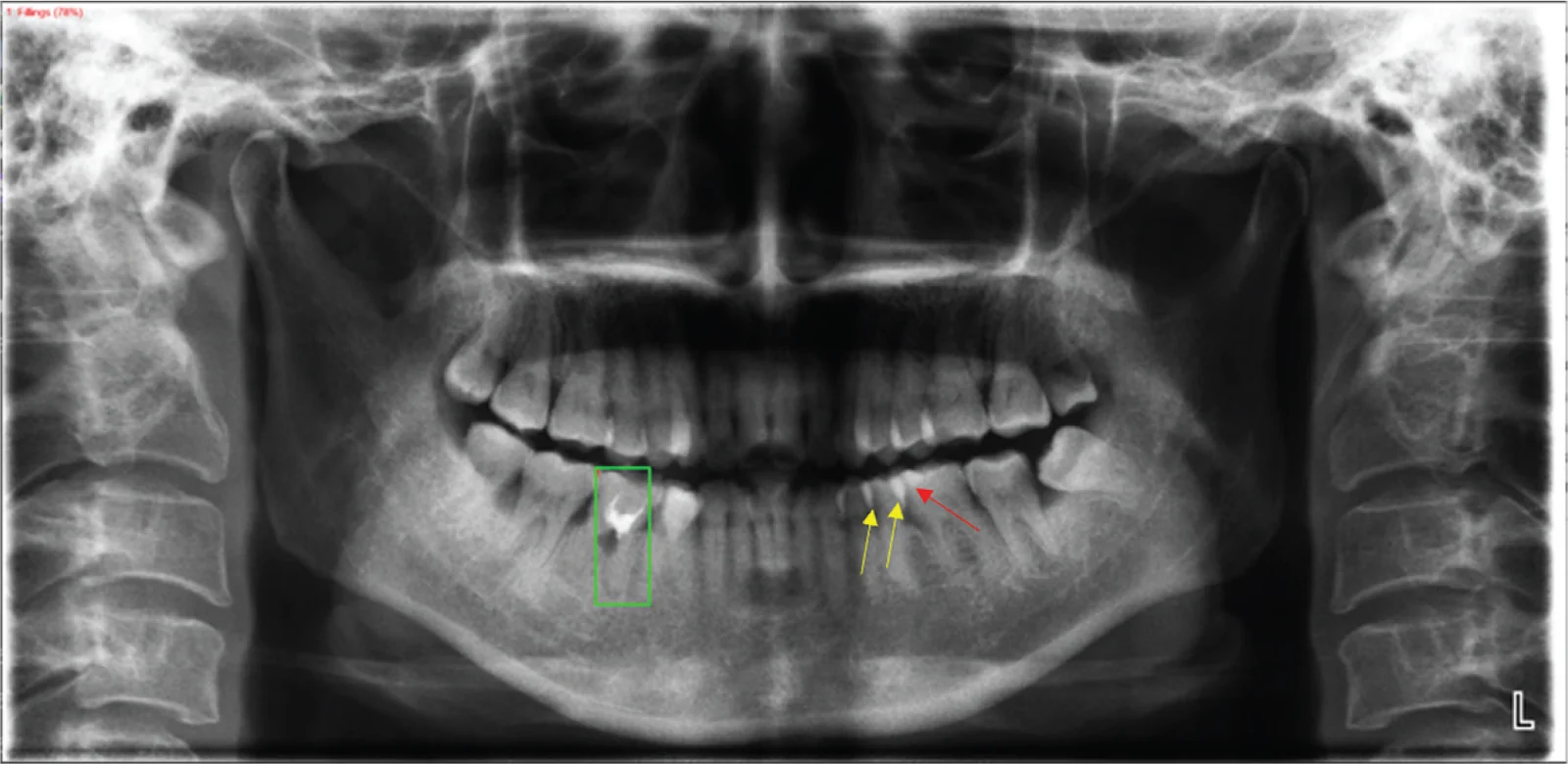
Analysis of a false-negative detection. The dental filling (red arrow) was missed by the model, likely due to overlapping enamel-induced highlights in the adjacent region (yellow arrows) that interfered with detection. The green bounding box indicates a model prediction on the adjacent tooth structure.

**Figure 3:**
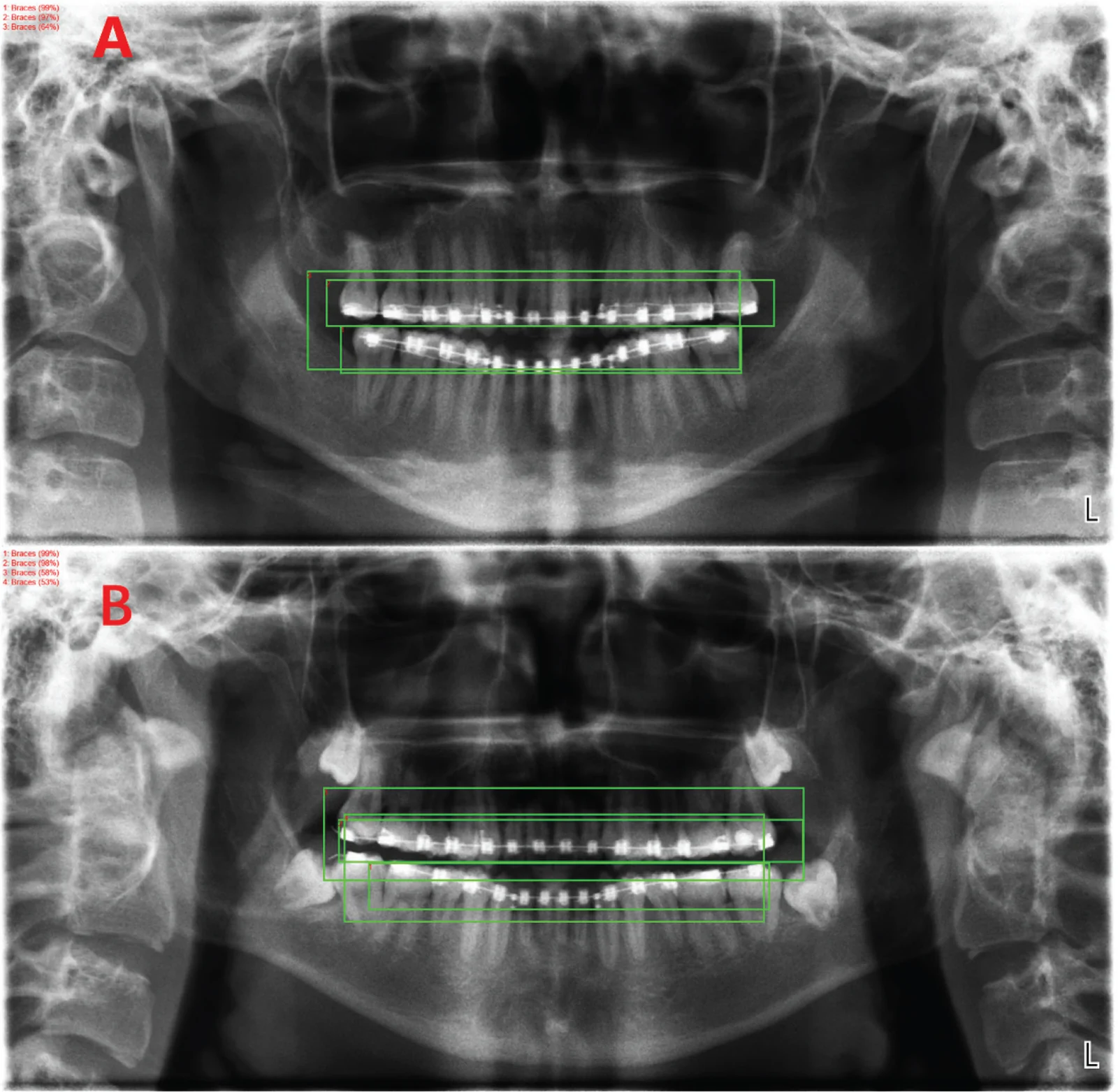
Overlapping false-positive detections for braces (Cases A & B) due to structural ambiguity with tooth contours/artifacts (precision = 0.32).

Table 5 summarizes the precision, recall, and F1-scores for all six dental restorations and appliances categories. Implants achieved the highest precision (0.93) and recall (0.92), attributed to their distinct metallic contrast and well-defined boundaries. Crowns demonstrated balanced performance (F1-score = 0.84), while fillings exhibited lower recall (0.56) due to size variability and overlapping enamel artifacts (Figure 2). Braces, despite high recall (0.89), suffered from low precision (0.32) caused by structural similarities to natural tooth contours and metal artifacts (Figure 3).

## Discussion

In recent years, the application of AI in oral medical imaging analysis has grown rapidly, yet its clinical utility continues to face significant challenges [16, 17]. Although prior studies have explored the potential of AI in single-task applications such as caries detection and implant localization [13], existing models generally exhibit three major limitations: (1) reliance on small-scale or noise-prone annotated datasets, leading to insufficient generalization capabilities [18]; (2) a severe mismatch between default parameters of generic object detection frameworks (e.g. faster R-CNN) and the scale distribution of densely packed small targets in dental imaging, resulting in persistently high false-negative rates [14, 19]; and (3) evaluation metrics predominantly focused on overall accuracy, lacking systematic validation of critical clinical performance aspects such as real-time processing and false-positive control [20]. To address these issues, this study proposes an object-detection framework evaluated on a clinically annotated dataset by integrating real-world clinical data, domain-adaptive model optimization, and multidimensional evaluation. To address these issues, this study proposes an object-detection framework evaluated on a clinically annotated dataset by integrating real-world clinical data, domain-adaptive model optimization, and multidimensional evaluation. This approach provides preliminary evidence that may inform the development of AI-assisted dental image analysis in oral medicine.

An important component of this study was the curation of a clinically annotated dataset comprising 2,434 anonymized panoramic X-ray images from the Radiology Department of the Affiliated Hospital of Qingdao University. Compared with many previous dental AI studies, this dataset provides a relatively larger sample for evaluating multiclass detection of dental restorations and appliances [21, 22]. Each image was annotated by trained dental professionals using Labelme, and inter-annotator agreement was assessed using mean IoU, which reached 0.92. These procedures were intended to reduce annotation inconsistency, a known challenge in medical image analysis [23, 24].

In this experimental setting, the enhanced dataset yielded numerically higher dataset-level metrics than the baseline dataset, with mAP@0.5 of 0.75 and AR@100 of 0.81. However, because the baseline and enhanced datasets differed in source, annotation scope, target categories, category definitions, and class composition, these results should be interpreted as descriptive findings from different dataset configurations rather than as a direct controlled estimate of performance improvement. Therefore, the observed differences may reflect dataset scale, annotation consistency, task-specific label design, and source-related characteristics and should be interpreted cautiously. These findings are consistent with previous reports suggesting that dataset scale, annotation quality, and task-specific label design can influence the performance of AI-based dental imaging systems [25, 26].

**Figure 4:**
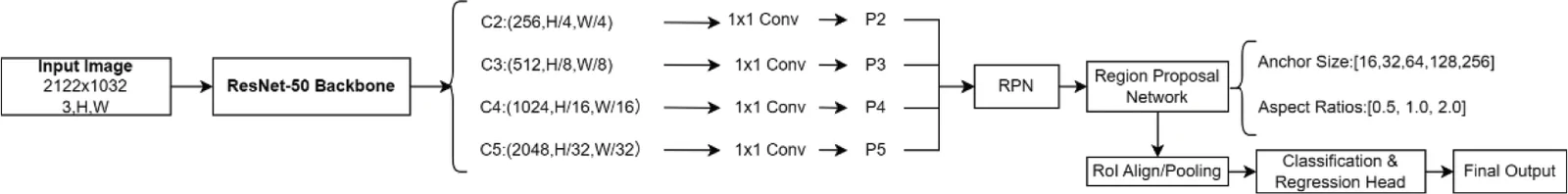
Illustration of faster R-CNN with FPN architecture highlighting lateral connections.

Recognizing the scale disparity between generic object detection frameworks and dental imaging characteristics, this study implemented domain-specific architectural refinements to the faster R-CNN pipeline. Empirical evidence indicates that default anchor sizes (32–512 pixels) in natural image detectors are suboptimal for identifying diminutive dental structures (< 5 mm) prevalent in panoramic radiographs [15]. This study implemented targeted optimizations to the faster R-CNN framework. By resizing anchor boxes from the default range for natural images (32–512 pixels) to 16–256 pixels, the model showed modest improvements in detection performance for localized dental targets, particularly fillings and root canal filling, as reflected by the class-wise results in Table 6. Leveraging the multiscale feature fusion capability of the ResNet-50-FPN backbone, the model was designed to improve feature representation in complex backgrounds (e.g. metal artifacts and anatomical overlaps), including challenging distinctions such as dental bridges and adjacent crowns (Figure 4). These refinements align with the anchor optimization strategy proposed by Raimundo et al. [27] for breast cancer lesion detection, underscoring the universal value of domain-adaptive design in medical imaging.

To comprehensively evaluate clinical applicability, this study not only focused on traditional accuracy metrics (e.g. mAP@0.5) but also incorporated multidimensional performance analysis. For instance, implants achieved optimal detection performance (precision = 0.93, recall = 0.92) owing to their high-contrast features, which may be relevant for future clinical applications. Conversely, braces suffered from substantial false positives (precision = 0.32) despite high sensitivity (recall = 0.89), primarily due to their curvilinear morphology mimicking natural dental arch contours – a diagnostic pitfall well-documented in orthodontic imaging studies [28]. This underscores the necessity for future work to embed anatomical priors (e.g. tooth segmentation masks) as proposed by Sivari et al. for restorations and appliances contextualization [29]. The average inference time per image was below 1.2 s when tested on a laptop workstation equipped with an Intel Core i9-14900HX CPU, 16 GB RAM, and an NVIDIA GeForce RTX 4060 Laptop GPU with 8 GB VRAM, suggesting potential workflow compatibility under similar computational conditions. However, additional validation is required before routine clinical implementation can be considered [20].

Despite significant advancements in multi-class restorations and appliances detection, this study remains subject to certain limitations. For example, fillings showed relatively low recall (0.56), and braces showed low precision (0.32) and a low F1-score (0.45), likely due to size variability, anatomical overlap, and metal-related artifacts. Future studies could address these challenges by incorporating three-dimensional imaging (e.g. cone-beam computed tomography, CBCT) or dynamic anchor generation algorithms [30, 31]. Furthermore, multicenter, multi-device external validation will be essential to ensure clinical generalizability and facilitate broader adoption. Future studies should further compare faster R-CNN with newer one-stage and transformer-based detection architectures on the same annotated dataset to determine the most suitable model for this specific dental imaging task.

In conclusion, we present a deep-learning pipeline for detecting dental restorations and appliances on panoramic radiographs. Using an expert-annotated single-center dataset, the model showed encouraging performance across categories, although performance remained variable for some classes. The findings support the feasibility of this approach within the present experimental setting. Further external, multicenter, and prospective validation is required to assess generalizability and clinical utility.
